# Development and Validation of a New Mouse Model to Investigate the Role of SV2A in Epilepsy

**DOI:** 10.1371/journal.pone.0166525

**Published:** 2016-11-18

**Authors:** Catherine Menten-Dedoyart, Maria Elisa Serrano Navacerrada, Odile Bartholome, Judit Sánchez Gil, Virginie Neirinckx, Sabine Wislet, Guillaume Becker, Alain Plenevaux, Priscilla Van den Ackerveken, Bernard Rogister

**Affiliations:** 1 GIGA-Neurosciences, University of Liège, Liège, Belgium; 2 Cyclotron Research Center, University of Liège, Liège, Belgium; 3 Neurology Department, CHU, Academic Hospital, University of Liège, Liège, Belgium; Montana State University Bozeman, UNITED STATES

## Abstract

SV2A is a glycoprotein present in the membranes of most synaptic vesicles. Although it has been highly conserved throughout evolution, its physiological role remains largely unknown. Nevertheless, Levetiracetam, a very effective anti-epileptic drug, has been recently demonstrated to bind to SV2A. At present, our understanding of the normal function of SV2A and its possible involvement in diseases like epilepsy is limited. With this study, we sought to develop a relevant model enabling analysis of SV2A’s role in the occurrence or progression of epilepsy. For this purpose, we generated a floxed *SV2A* mouse model with conditional alleles carrying LoxP sites around exon 3 by means of a gene-targeting strategy. The SV2A lox/lox mouse line is indistinguishable from wild-type mice. When the recombination was observed in all cells, a model of mice with both *SV2A* alleles floxed around exon 3 recapitulated the phenotype of SV2A KO mice, including seizures. However, the specific invalidation of *SV2A* in the CA3 hippocampal region was not followed by epileptic seizures or decrease in the epileptic threshold on *pentylenetetrazol (PTZ)* test. These results demonstrate that the floxed SV2A mouse line has been successfully established. This transgenic mouse model will be useful for investigating SV2A functions related to cell types and developmental stages.

## Introduction

The synaptic vesicle protein 2 (SV2) family comprises transmembrane glycoproteins in three paralogs: SV2A, SV2B, and SV2C. These are found in the membranes of neuronal synaptic vesicles and in secretion vesicles of endocrine cells [1]. These proteins contain a highly-glycosylated backbone measuring 80kDa. They possess 12 transmembrane regions (TMRs) with N- [2] and C-terminal cytoplasmic sequences and a large intravesicular loop. Comparisons of the different SV2 homologs have revealed that TMRs and cytoplasmic loops are highly conserved, while the intravesicular loop exhibits little homology. Moreover, all SV2 proteins are N-glycosylated. In normal adult brains, SV2A is the only form ubiquitously expressed in all brain areas, with the possible exception of the trigeminal and facial nuclei [1]. SV2A is also expressed in the neuroendocrine cells [1, 3] and at the neuromuscular junction [4]. SV2B is primarily expressed in the cerebral cortex and hippocampus [1], while SV2C expression is much more restricted to a few specific areas, including the striatum, the substantia nigra, and the hippocampus [5, 6].

Although SV2A is a highly-conserved sequence across all of evolution [7], with a well-documented expression profile, its physiological role remains elusive. However, studies have indicated its involvement in several processes, such as regulating synaptic vesicle exocytosis and maintaining calcium homeostasis in synaptic vesicles [2, 8]. More recently, SV2A was identified as the specific binding partner for Levetiracetam, a second generation antiepileptic drug. Levetiracetam reduces presynaptic glutamate release and is able to modify the natural history of epilepsy in various rodent models [9].

To better characterize the role of SV2A, SV2A-knockout (SV2A KO) mice have been engineered. Homozygous SV2A- (-/-) KO mice exhibited lethal seizures at P7 and died quickly after birth, at P15 [2, 10]. Heterozygous SV2A (+/-) mice, however, had a normal lifespan [11]. The brain morphology of these mice appeared unchanged [10]. At the functional level, spontaneous inhibitory postsynaptic current (sIPSC) frequencies were found to be reduced in the CA1 and CA3 regions of the hippocampus of SV2A- (-/-) deficient mice. However, miniature inhibitory postsynaptic current (mIPSC) appeared unchanged, meaning that the protein machinery allowing the vesicles filled with GABA is not affected but the coupling molecular machinery between the action potential reaching the synaptic button and the GABAergic-dependent neurotransmission is. [10, 12].

As SV2A-KO models appear unsatisfactory to fully characterize SV2A functions in adult brains, we decided to generate Cre-Lox-based conditional SV2A mice with SV2A silenced in specific brain areas. We herein report our construction of mice with SV2A alleles floxed around exon 3. Once this recombination was observed in all cells, we could recapitulate the phenotype of SV2A-KO mice. We also report the specific deletion of SV2A in the CA3 hippocampal region of adult mouse brains, demonstrating that the disappearance of SV2A from the CA3 region in these conditions do not lead to the onset of epileptic seizures or a decrease in the epileptic threshold, based on pentylenetetrazol (PTZ) test.

## Methods

### Animals

SV2A flox/flox mice (generated by Ozgene^®^) were crossed with either Ubiquitin-creERT2 (Ubc-cre) (Jackson Laboratory^®^ #007001) or Grik4-Cre mice (Jackson Laboratory^®^ #006474). With the former, we obtained Ubc-Cre/+; SV2A flox/flox pups (hereafter referred to as “Ubc:SV2A-cKO”) and Ubc+/+, SV2A flox/flox pups. When crossed with Grik4-Cre mice (Jackson Laboratory^®^ #006474), we obtained Grik4-Cre/+; SV2A flox/flox mice (hereafter “Grik4:SV2A-cKO”) and Grik4+/+, SV2A flox/flox pups with a C57BL/6 genetic background. We assessed Grik4:Cre recombinase activity by means of the RosaR26R-Yellow fluorescent protein (eYFP; R26R-YFP) (Jackson Laboratory^®^ #006148) reporter mouse line. The recombination in the latter led to permanent expression of eYFP. Ubc+/+, SV2A flox/flox, and Grik4+/+, as well as SV2A flox/flox mice were all labeled “wild-type” (WT) as they did not display abnormal phenotypes.

Ubc:cre-ERT2 recombinase activity was induced by Tamoxifen (Sigma- Aldrich^®^, Belgium), dissolved at 30mg/*mL* in a sunflower oil/ethanol (9:1) mixture, then administered at E18.5 to timed-pregnant mice by oral gavage (*180mg/Kg* body weight/administration).

Animal care was in accordance with the declaration of Helsinki and the guidelines of the Belgium Ministry of Agriculture, in agreement with the European Community laboratory animal care and use regulations. Experimental research on the animals was performed with the approval of the University of Liège ethics committee (Belgium), filed under numbers 1258 and 1753, accepted in 2011 and 2016, respectively.

### Genomic Design, Manipulation, and *SV2A*^lox/lox^ Mouse Generation

mRNA/cDNA sequences corresponding to the *SV2A* gene were identified on the National Center for Biotechnology Information database; GenBank accession number NM_022030 and BC046587. FLSniper plasmid was used to construct the targeting vector. This vector was built using three fragments: the 3’ and 5’ homology arms flanking exon 3 of the *SV2A* gene, generated by polymerase chain reaction (PCR) from C57BL/6 genomic DNA, and the LoxP arm. PGK-Neo-pA-SD-IS was used as a selection cassette, containing the exon 3 sequence flanked by LoxP sites by homologue recombination. This cassette was then inserted upstream of *SV2A*’s exon 3 of the targeting vector. The required sequence was contained within bacterial artificial chromosome (BAC) clones RP23-171B14 and RP23-16I15. Intron/exon structure was confirmed by aligning the mouse mRNA sequence (NM_022030) with the Ensembl^®^ chromosome 3 sequence. Clones with the correct insertion were screened and confirmed by PCR, then linearized and electroporated in embryonic stem-cell (ES cell) lines. Notice that the PGK-neo cassette is flancked by FRT sites and can be deleted using FLPe recombinase. Then following southern blotting, homologous recombinant ES cells were injected into C57BL/6J blastocysts. Chimeric mice were crossbred with C57BL/6J WT mice to obtain germline transmission.

### Genotyping

DNA was extracted from the mice’s tail tips. Tail samples were digested in buffer solution (0.1M Tris-HCl, 5mM EDTA, 0.2M NaCl, and 0.2% sodium dodecyl sulfate [SDS]) containing proteinase K (*16ug/mL* Promega^®^) overnight at 55°C. Following precipitation with isopropanol, the DNA was washed in a 70% ethanol solution and then suspended in water. PCR was performed using the following primers (Integrated DNA Technologies, Belgium) and cycles: (i) *SV2A* gene amplification: SV2A^lox/lox^ forward: 5’–TGG GTT GGG CTA CTG TTA GG– 3’, SV2A^lox/lox^ reverse: 5’–AGT TGG GAA GGA GGC AAG AT– 3’, 35 cycles: 94°C 30sec, 52°C 30sec, 72°C 1min30; (ii) Cre detection: Cre forward: 5’–GCG GTC TGG CAG TAA AAA CTA TC– 3’, Cre reverse: 5’–GTG AAA CAG CAT TGC TGT CAC TT– 3’, Internal control forward: 5’–CTA GGC CAC AGA ATT GAA AGA TCT– 3’, Internal control reverse: 5’–GTA GGT GGA AAT TCT AGC ATC ATC C– 3’, 35 cycles: 94°C 30sec, 52°C 30sec, 72°C 1min30.

### Tissue Harvesting and Sections

Mice aged 8 to 12 weeks old were anesthetized using 60mg pentobarbital (Ceva Sante Animal^®^), then perfused intracardially with 0.9% NaCl followed by 4% ice-cold paraformaldehyde (PFA) solution (in 0.1M phosphate-buffered saline, [PBS]). The mouse brains were then harvested and post-fixed for two hours at 4°C in 4% PFA solution, and finally cryopreserved overnight in 30% sucrose and 0.1M PBS. The brains were frozen in isopentane for preservation at -20°C prior to being sliced into 20μm-thick cryosections.

### Immunolabeling

The hippocampus cryosections were first washed in 0.1M PBS then permeabilized and blocked for 30 minutes with 5% normal donkey serum (NDS) and 0.3%triton X-100 (Sigma-Aldrich^®^) in 0.1M PBS at room temperature. The same solution containing the primary antibody–(chicken anti-GFP antibody [1/1000, ABCAM^®^, ab6556] was incubated overnight at 4°C. After three washes in 0.1M PBS, the sections were incubated for 90 minutes with a FITC-conjugated secondary antibody (1/500 Jackson Immunoresearch Laboratories^®^). After three washes in 0.1M PBS, the sections were incubated with Hoechst (1/5000) for 10 minutes, then dried and mounted in Safe Mount. Image acquisition was performed using a Zeiss Axiovert 10VR microscope (Carl Zeiss^®^) coupled with FluoView software (Olympus^®^).

### *In Situ* Hybridization

Following post-fixation with 4% PFA, the hippocampus cryosections were treated with 0.25% acetic anhydride in 0.1 M triethanolamine. The sections were washed with 0.1M PBS and prehybridized for 2h with 50% formamide, 5 × sodium saline citrate buffer (SSC), and citric acid to pH with 6.1% SDS, 500μg/mL yeast RNA, hybridization cocktail, and 50% formamide (Lucron bioproducts^®^, Belgium). The 5’-digoxigenin-labeled LNA probes (30nM, final) were added to the fresh hybridization cocktail and hybridization occurred overnight at 55°C.

The LNA probe /5DIGN/ TGACTGAGAGTGAGATGAGCAGA/3DIG_N/ (Exiqon^®^) was designed to target exon 3 of *SV2A* in order to distinguish WT from Grik4:Sv2A-cKO and a scramble LNA probe was used as a control. The hybridized sections were then washed twice with 50% formamide, 2 × SSC, and 0.1% tween at 50°C, followed by a single wash in buffer B1 (150mM NaCl, 100mM Tris, pH 7.4) at room temperature (RT). The sections were then treated for 1h at RT in buffer B1 + 10% normal goat serum (buffer B2), followed by overnight incubation at 4°C with alkaline phosphatase (AP)-labeled anti-digoxigenin antibody (1:2000; Roche Applied science^®^, Belgium) in buffer B2. After extensive washes with PBS containing 0.1% Tween 20, the sections were exposed to the substrate for AP, nitroblue tetrazolium, and 5-bromo-4-chloro-3-indoyl phosphate (NBT/BCIP; Sigma–Aldrich^®^). Reactions were blocked by washes with PBS, followed by postfixation in 4% PFA for 20 minutes, then finally rinsed in MilliQ water. The slides were cover-slipped with Aquamount^TM^ (Merck^®^, Germany).

### *In Vitro* Autoradiography

*In vitro* autoradiography was performed in the Grik4:SV2A-cKO and WT mice aged 8 to 12 weeks. Following brief isoflurane inhalation, the mice were decapitated for careful removal of their brains, which were immediately frozen in 2-methylbutane cooled with dry ice (-29°C). Coronal sections (20-μm thick) were cut across the hippocampus using a -20°C cryostat (CM3000, Leica^®^) then thaw-mounted on glass slides and allowed to air-dry prior to storage at -80°C until use. The day of the radiotracer synthesis experiment, the slides were left to reach room temperature then incubated for 20 minutes in Tris PBS (138mM NaCl, 2.7mM KCl, pH adjusted to 7.5) containing 9.25kBq/mL (0.25μCi/mL) of ^18^F-UCB-H. Following incubation, the slides were dipped in cold buffer (4°C) for 90 seconds, in distilled cold water (4°C) for 90 seconds, then dried and placed on phosphor imaging plates for 90 minutes (Kodack^®^). All films were analyzed by a computer-assisted image analysis system (Personal Molecular Imager, BIORAD^®^). At the end of the autoradiography protocol, the brain slices were stained with a classic eosin-hematoxylin procedure and the resulting anatomical images digitalized. We used PMOD software to co-register the autoradiographic images with anatomical images and then analyze the data. Regions of interest (ROIs) were manually drawn on the anatomical images to segment the different hippocampus areas (the dentate gyrus and Ammon’s horn CA1, CA2, and CA3) according to a mouse brain atlas. Radiotracer labeling intensity in each ROI was extracted, the results expressed in Radioluminescence Optical Density (ROD).

### Micro-Dissection

Cryosections (40μm thick) were first extracted from non-perfused mouse brains of 8-12-week-old mice. Micro-dissection of regions of interest was then performed using an LMD7000 laser microdissecter (Leica-Microsystems^®^), recovered either in PBS or in lysis buffer (Triton X-100, 0.1M PBS, 1.5M NaCl, 0.5M EDTA) for DNA or protein extraction, respectively. DNA extraction was performed using a QIAamp DNA Mini kit (Qiagen^®^) according to the manufacturer’s instructions. Protein extraction was performed as described below.

### Protein Extraction and Western Blotting

Briefly, the tissues were ground in lysis buffer (Triton X-100, 0.1M PBS, 1.5M NaCl, 0.5M EDTA) and incubated on ice for 15 minutes. Following centrifugation at 10,000 x g, 4°C, for 10 minutes, the supernatant was collected then conserved at -80°C. Following protein concentration quantification, 25μg of total extracted protein were diluted in 20μl of loading buffer (106mM Tris, 141mM Tris base, 2% LDS, 10% Glycerol, 0.51mM EDTA, 0.22mM SERVA Blue G250, and 0.175mM red Phenol, pH: 8.5) and heated at 70°C for 10 minutes. Proteins were then separated through a 12% acrylamide commercial gel (4–12% NuPage, Life Technologies^®^), in MOPS buffer (50mM Tris Base, 0.1% SDS, and 1mM EDTA, pH 7.7), for 15 minutes at 80V then 90 minutes at 150V. Proteins were transferred onto a PVDF membrane (Roche^®^) using a semi-dry transfer system (Biorad^®^) for 2h at 50V in transfer buffer (25mM Tris Base, 192mM glycine, and 20% methanol). The PVDF membrane was then washed three times in TTBS (50mM Tris Base, 150mM NaCl, 1M HCl, 0.1% Tween 20, pH 7.5) and blocked for 1h at RT in TTBS with 5% milk. We then incubated it overnight with SV2A antibody (1:2000, Abcam^®^) diluted in blocking buffer at 4°C. Following three washes, the membrane was incubated with a secondary antibody (anti-Rabbit HRP, 1/5000, Abcam^®^) diluted in blocking solution, for one hour at RT. For quantification, anti-β-actin-peroxydase antibody was added (1:10,000, Sigma^®^) during one hour at RT after three 5 min washes of TTBS. Finally, after three additional washes, it was incubated with ECL substrate (Thermo Fischer Scientific^®^) and chemiluminescent signals were acquired by means of ImageQuant camera and software (GE Healthcare^®^).

### RNA Extraction and Quantitative Real-Time PCR (qRT-PCR)

Total RNA was extracted from the DG, CA3, and CA1 areas using TRIzol reagent (Invitrogen^®^), treated with DNase I (Promega^®^) prior to reverse transcription, according to the manufacturer’s instructions. First-strand cDNA was synthesized from 1μg total RNA by means of M-MLV Reverse Transcriptase (Promega^®^). The resulting cDNA product was used for real-time PCR in a LightCycler 480 (Roche^®^) using a SYBR Green Master Mix (Roche^®^). Gene expression levels were normalized upon the glyceraldehyde-3-phosphate dehydrogenase (GAPDH) housekeeping gene. All amplifications were performed in triplicate and a minimum of three biological replicates were performed. The primer sets purchased from IDT^®^ were: SV2A, forward GTCTTTGTGGTGGGCTTTGT, reverse CGAAGACGCTGTTGACTGAG; SV2B, forward AAACGCGGTGAGCATCTTAG, reverse ACTTCAGAGCCACCATGGAC; SV2C, forward TCTTTGCCTTCCTCTCCTCA, reverse GAACATGCAGAGCCAACTGA.

### PTZ Test

The PTZ test (Sigma-Aldrich®) was performed following the recommendations laid down by Suzuki *et al*. [13]. PTZ was freshly dissolved in sterile PBS and administered by intraperitoneal injection at a dose of *50mg/Kg*, to an ultimate volume of 200–300μl. The mice were observed for 10 minutes post-PTZ injection and seizure onset was measured according to the PTZ scale described by Lüttjohann. This scale defines six stages of seizure intensity: sudden behavioral arrest (Stage 1), facial jerking (Stage 2), neck jerks (Stage 3), clonic seizures (sitting) (Stage 4), tonic seizure (lying on belly) (Stage 5), and tonic seizure with wild jumping (Stage 6).

For a positive control, we injected PTZ into two *SV2A*-hemizygous mice (SV2A+/-), which died less than 10 minutes after the injection from convulsions and tonic-clonic seizures. Statistical analysis was performed by a one-way analysis of variance (ANOVA), and the level of significance was set at 0.05.

### Statistical Methods

All data is reported as mean ± standard error of the mean (SEM). Statistical analyses were performed using GraphPad InStat software (GraphPad). Student’s *t*-tests were used for paired comparisons and one-way ANOVA, followed by Tukey’s post-hoc test, for multiple comparisons. The level of significance was set at * *p* <0.05, ** *p* <0.01, ***p<0.001.

## Results

### Generation of *SV2A*-Conditional Mice

**Fig 1:**
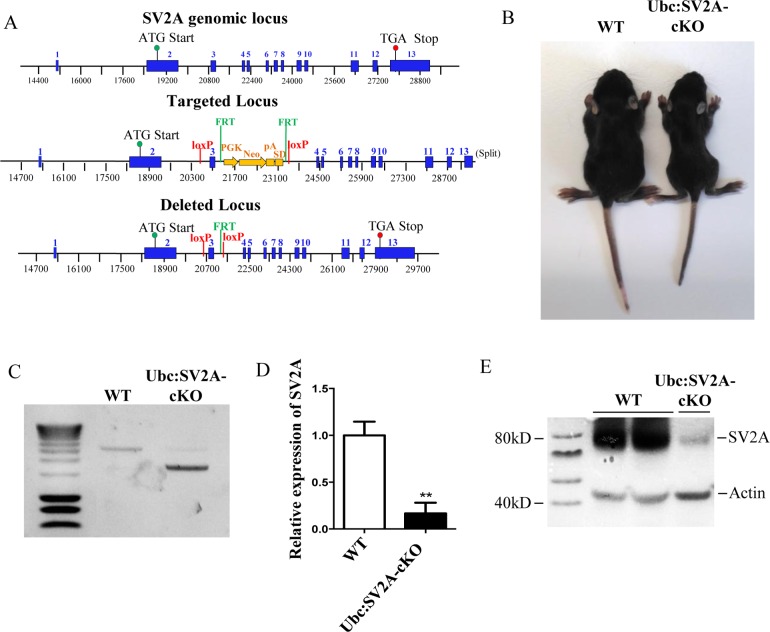
Conditional removal of the *SV2A* exon 3 in Ubiquitin-positive cells led to seizure. A: Illustration of the homologous recombination strategy used to generate the *SV2A* lox/lox allele. The *SV2A* gene is located on chromosome 3, composed of 13 exons. A PGK-Neomycin selection cassette flanked by FRT sites was inserted upstream of exon 3. Following FLP recombinase, the *SV2A* gene contained the exon 3 flanked by LoxP sites that could be deleted using Cre-recombinase. B: Comparison of P12 WT and conditional SV2A-knockout (Ubc:SV2A-cKO) littermate mice. Note the reduced size of the Ubc:SV2A-cKO mice. C: PCR genotype analysis of WT and Ubc:SV2A-cKO mouse brains (the loxed allele at 2,500bp and recombined allele at 1,500bp). D: SV2A mRNA expression in brains, measured by quantitative real-time PCR from WT and Ubc:SV2A-cKO mice. The level of SV2A mRNA was normalized using the level of GAPDH mRNA. All data is presented as mean ± SEM, ** *p* <0.01. E: Western blot analyses showing the depletion of SV2A protein in Ubc:SV2A-cKO brains. Actin was used as a control for the proteins loaded in each protein extract.

To generate mice with *SV2A* conditional deletion, we used a Cre/loxP recombination system. In mice, the *SV2A* gene is located on chromosome 3 (Ensembl Gene Report ID: ENSMUSG00000038486) and has 13 exons spread over approximately 14kb. A PGK-Neomycin selection cassette was inserted upstream of exon 3 to select ES cell containing this construction of interest (Fig 1A). At the end, the *SV2A* gene contained the exon 3 flanked by LoxP sites that could be deleted using Cre-recombinase (Fig 1A). Stem cells containing the floxed gene were then injected into C57BL/6 blastocysts. Chimeric mice were then crossed with C57BL/6 wild-type (WT) mice to obtain SV2A^lox/lox^ homozygote mice. When a recombination occurred, the exon 3 deletion introduced an open reading frame shift and produced an early STOP codon that disrupted, at the protein level, the transporter/Major Facilitator Superfamily domain. Wild-type, floxed, and recombined SV2A alleles were genotyped using PCR, which allowed us to clearly identify floxed (2500bp), wild-type (2000bp), and recombined (1500bp) alleles (data not shown). The mice that were homozygous for the floxed allele were viable, fertile, and exhibited no obvious phenotype (data not shown).

### Ubiquitous Recombination of SV2A^lox/lox^ Led to Significantly Reduced *SV2A* Production and Mimiced the SV2A KO Phenotype

To determine whether SV2A flox/flox mice exhibited efficiently-invalidated *SV2A* expression, we crossed SV2A^lox/lox^ mice with Ubiquitin-CreERT2 mice to generate pups (*i*.*e*. Ubc: SV2A-cKO). We assessed SV2A expression in brain sections extracted from pups, at postnatal stage 12 (P12), that had received tamoxifen during embryonic stages 18.5 (E18.5). The pups disrupted for *SV2A* exhibited spontaneous seizures at approximately P12. The mice that experienced epileptic seizures were smaller and thinner compared to the WT mice (Fig 1B). Following sacrifice, *SV2A* gene recombination was revealed by genotyping the DNA extracted from brain extracts (Fig 1C) and tail biopsies (data not shown). As expected, quantitative RT-PCR performed on the RNA brain extracts demonstrated massively reduced SV2A mRNA levels in Ubc:SV2A-cKO mice versus their WT littermates (Fig 1D) (**: *p* <0.01). To confirm the depletion of SV2A at the protein level, we performed western blotting on brain protein extracts, again observing significantly reduced SV2A protein expression in the Ubc:SV2A-cKO mice versus the WTs (Fig 1E). Altogether, these results demonstrated that the *SV2A*^lox/lox^ gene is efficiently recombined by Cre-recombinase, and that the deletion of the *SV2A* gene’s exon 3, occurring in late embryonic development (E18.5), could mimic the SV2A KO phenotype.

### Validation of *SV2A* Gene Recombination in the Hippocampus of Grik4-Cre/SV2A^lox/lox^/ROSA-EYFP Mice

**Fig 2:**
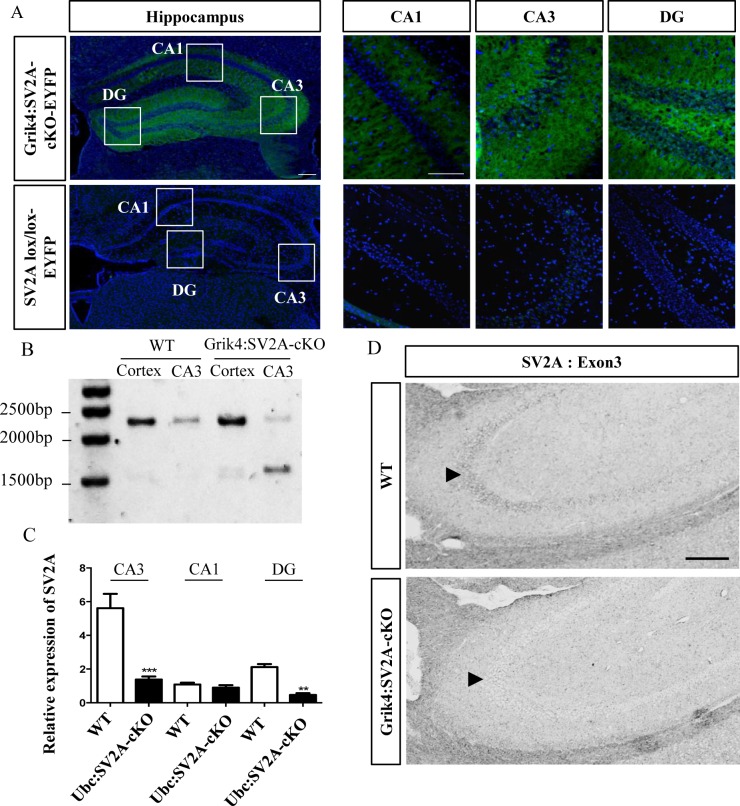
Specific invalidation of *SV2A* in the CA3 and DG areas of the adult hippocampus. A: Cre-mediated EYFP reporter expression demonstrating Cre recombination in the CA3 and DG areas of the hippocampus of Grik4-CRE/SV2A^Lox/Lox^/-ROSA-EYFP mice, versus controls (*i*.*e*.: SV2A^lox/lox^/ROSA-EYFP) (Green: e-YFP; blue: Hoechst; scale bar = 250μm). B: PCR genotype analysis on DNA extracted from the CA3 region or cerebral cortex and microdissected, from WT and Grik4:SV2A-CKO mouse brains (the loxed allele amplicon migrated at 2,500bp and the recombined allele amplicon at 1,500bp). C: SV2A mRNA expression in the microdissected hippocampus (CA3, CA1, DG), measured by quantitative real-time PCR, from WT and Grik4:SV2A-cKO mice. The SV2A mRNA level was normalized using the GAPDH level. The data is presented as mean ± SEM, ** *p* <0.01, ***p <0.001. D: Conditional removal of *SV2A* from adult mouse CA3 areas, validated by *in situ* hybridization using an LNA probe targeting an exon 3 sequence of the *SV2A* transcript. The arrowhead indicates the hippocampus. Scale bar = 500μm.

Previous studies have demonstrated the relationship between seizures and SV2A depletion. Moreover, *SV2A*–null mice have been reported dying at approximately P15 in status epilepticus [2, 10]. With the aims of bypassing early lethality and analyzing *SV2A*’s functions in epilepsy, we thus established a conditional mouse line carrying *SV2A* deletion in the hippocampus, starting after P14 or, in other words, after the earliest and main postnatal stages of neurodevelopment. For this purpose, we crossed *SV2A*^*lox/l*ox^ mice with Grik4-Cre^+/-^ mice [14] that expressed the Cre recombinase controlled by the glutamate receptor ionotropic kainate 4 (*Grik4*) gene promotor (Grik4:SV2A-cKO). In these mice, Cre/loxP recombination was described manifesting at approximately P14 in the CA3 hippocampus area [14]. Later, at P56, recombination had occurred in nearly 100% of the CA3 pyramidal cells [14]. To assess the Cre recombinase activity, we first crossed the Grik4:SV2A-cKO line with a R26R-YFP reporter mouse line, in which the recombination led to permanent expression of YFP (Grik4-Cre^+^/SV2A^lox/lox^/ROSA-EYFP). As expected, the Grik4-Cre^+^/SV2A^lox/lox^/ROSA-EYFP mice specifically expressed YFP in the entire hippocampus, although only the CA3 and dentate gyrus (DG) cell bodies were EYFP-positive (Fig 2A). It should be noted that the recombination also occurred in the cortex and hypothalamus neurons, though at lower frequencies (data not shown).

To assess the specificity of the *SV2A* recombination in the hippocampus CA3 region, we micro-dissected this region along with the cerebral cortices and performed PCR, following DNA extraction. As expected, the *SV2A* recombination was only observed in the CA3 region of Grik4:SV2A-cKO mice, as opposed to those of WT animals and the cerebral cortex samples (Fig 2B). We then analyzed the SV2A mRNA expression profile in the hippocampus by performing qRT-PCR on RNA extracted from the micro-dissected CA1, CA3, and DG regions of GriK4:SV2A-cKO mice, compared to those of WTs. Interestingly, the highest level of SV2A expression was the CA3 in WTs. Significant levels of SV2A expression were also seen in the DG and, to a lesser extent, CA1. When RNA was extracted from these micro-dissected regions from the hippocampus of GriK4:SV2A-cKO mice, they also revealed a statistically-significant decrease in SV2A mRNA expression in the CA3 area (***: *p* <0.001) and DG (**: *p* <0.01) (Fig 2C), while the expression in the CA1 region remained the same. These results were confirmed by *in situ* hybridization using an LNA probe targeting an exon 3 sequence of *SV2A*, proving the strong reduction of SV2A mRNA expression in the CA3 hippocampus area of Grik4:SV2A-cKO mice in comparison with that of the WTs (Fig 2D). All in all, these findings provide evidence of a specific disruption of the *SV2A* gene leading to a decrease in its mRNA expression in the CA3 and DG hippocampus areas of Grik4:SV2A-cKO mice.

**Fig 3:**
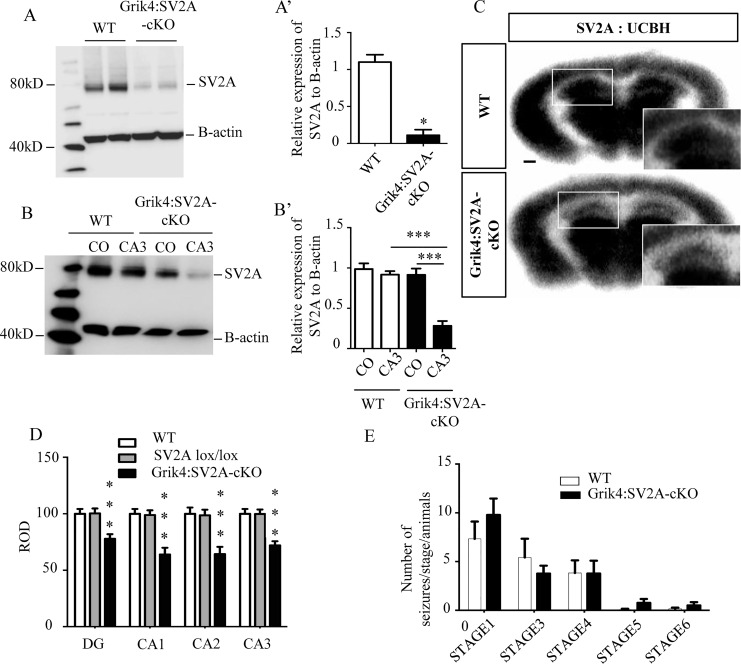
The absence of SV2A protein in the hippocampus does not lower the epileptic threshold. A: Western blot analyses revealing SV2A protein depletion in the hippocampus of Grik4:SV2A-cKO mice in comparison with that of WTs. A’: Quantification of the SV2A proteins extracted from the hippocampus of WT and Grik4:SV2A-cKO mice (n = 3/group, data presented as mean ± SEM, ***p <0,001). B: Western blot analyses demonstrating SV2A protein depletion in the cerebral cortex and CA3 of Grik4:SV2A-cKO mice compared to WT. B’: Quantification of the SV2A proteins (n = 3/group, data presented as mean ± SEM, ***p <0,001). C: Conditional removal of *SV2A* from adult mice CA3 areas validated by using *in vitro* autoradiography with the radioligand ^18^F-UCB-H (scale bar = 500μm). D: Semi-quantitation of ^18^F-UCB-H signal intensity in the hippocampus (ROD: radioluminescence optical density). E: Number of seizures per animal in those expressing different behavioral categories post-PTZ injection: sudden behavioral arrest (Stage 1), facial jerking (Stage 2), neck jerks (Stage 3), clonic seizures (sitting) (Stage 4), tonic seizure (lying on belly) (Stage 5), tonic seizure with wild jumping (Stage 6).

To determine if SV2A protein was still present in the CA3 pyramidal cells of Grik4:SV2A-cKO mice, we performed western blotting on whole hippocampus protein extracts. SV2A expression levels significantly decreased in the hippocampus of the Grik4:SV2A-cKO mice, compared to that of the WTs (***: *p* <0.0001) (Fig 3A and 3A’). To assess the specificity of the SV2A protein depletion in the CA3 hippocampus region, we micro-dissected this region and the cerebral cortex of adult mice (*i*.*e*., Grik4:SV2A-cKO and WT) and performed western blotting. We detected a similar amount of SV2A protein in the CA3 hippocampus region and cerebral cortex of the WT animals. On the other hand, as expected, we observed a stronger reduction of SV2A protein signal in the CA3 hippocampus region than in the cortex in Grik4:SV2A-cKO animals (***: *p* <0.001) or the CA3 hippocampus region of the WTs (***: *p* <0.001)(Fig 3B and 3B’). These results were confirmed by *in vitro* autoradiography using the ^18^F-UCB-H radioligand. This radiotracer has been shown to specifically bind to the SV2A protein [15, 16]. Autoradiograms obtained post-incubation using a constant radiotracer concentration enabled us to visualize decreased binding of ^18^F-UCB-H in all hippocampus regions of the Grik4:SV2A-cKO animals compared to WT littermates (***: *p* <0.001) (Fig 3C and 3D). Significantly, the signal was roughly the same in the other brain regions. Altogether, these results demonstrated the specific recombination of the *SV2A* gene in the hippocampus, primarily in the CA3 area, of Grik4: SV2A-cKO mice and, consequently, the strong decrease of SV2A protein in the CA3 region of these animals.

To assess the efficiency of the recombinaison, we evaluated the loss of SV2A protein according to mRNA expression in Grik4:SV2A-cKO and Ubc:SV2A-cKO mouse line. Both mouse lines reduced more than half of mRNA and protein expression (S1A Fig). Interestingly, we observed in Ubc:SV2A-cKO cortex a depletion of SV2A mRNA expression greater than the decrease in protein (S1B Fig). On the other hand, we detected a similar amount of SV2A protein and mRNA in CA3 of the hippocampus of Grik4:SV2A-cKO. This result suggest that both mouse lines are equally efficient to invalidate SV2A expression.

### Grik4:SV2A-cKO Adult Animals Exhibited Neither Spontaneous Seizures nor Lowered Epileptic Thresholds

**Fig 4:**
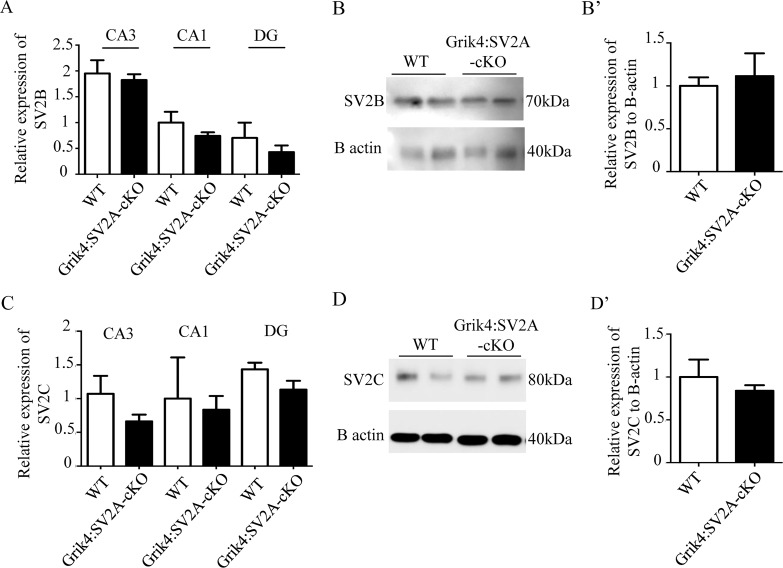
SV2B and SV2C are not upregulated in the absence of SV2A in the hippocampus. A: SV2B mRNA expression in the hippocampus of WT and Grik4:SV2A-cKO mice, measured by quantitative real-time PCR. The SV2A mRNA levels were normalized using the GAPDH mRNA levels. B: Western blot analyses showing the expression of SV2B protein in the hippocampus of Grik4:SV2A-cKO mice, versus that of WT mice. B’: Quantification of the SV2B proteins extracted from the hippocampus of WT and Grik4:SV2A-cKO mice. C: SV2C mRNA hippocampus expression in WT and Grik4:SV2A-cKO mice, measured by quantitative RT-PCR. D: Western blot analyses showing the expression of SV2C protein in Grik4:SV2A-cKO mouse hippocampus versus that of WT mice. D’: Quantification of the SV2C proteins extracted from the hippocampus of WT and Grik4:SV2A-cKO mice. (n = 3/group, data presented as mean ± SEM)

The Grik4:SV2A-cKO mice developed normally; viable and fertile with no obvious phenotype, such as epileptic seizures or increased mortality, compared to the WT littermate animals (data not shown). As the *SV2A*-null heterozygous mice did not exhibit specific problems, yet had reduced epileptic thresholds [17], we explored the possibility that the Grik4:SV2A-cKO mice could present the same phenotype. We therefore injected adult mice with pentylenetetrazole (PTZ) and analyzed seizure occurrence by means of the PTZ scale, as previously described [13]. Briefly, the PTZ scale defines six stages of seizure onset induced by PTZ injections, from frozen behavior (Step 1) to convulsions (Step 6). No significant difference was observed between the groups (WT and Grik4:SV2A-cKO mice) throughout the six stages of the scale (Fig 3E). We thus concluded that the adult Grik4:SV2A-cKO mice were neither subject to spontaneous epileptic seizures nor characterized by lower epileptic thresholds, despite presenting reduced levels of hippocampal SV2A protein. Interestingly, in human epileptic foci, the down-regulation of SV2A expression is associated with an overexpression of SV2C [18]. We thus investigated if the levels of other proteins of the SV2 family were affected while SV2A is strongly reduced. Using RT-PCR and western blot analyses, we measured the levels of SV2B (Fig 4A, 4B and 4B’) and SV2C (Fig 4C, 4D and 4D’) expression in the hippocampus of WT and GriK4:SV2A-cKO mice. No significant difference was observed between the groups (WT and Grik4:SV2A-cKO mice) for SV2B (Fig 4A, 4B and 4B’) or SV2C expression (Fig 4C, 4D and 4D’). In all, these results excluded the possibility of a compensatory phenomenon relating to a SV2A-depleted hippocampus.

## Discussion

Although the exact role of the SV2A protein is still unclear, it is abundantly present in the synaptic vesicles of all presynaptic nerve terminals, as well as in most neuroendocrine secretory granules [1, 3, 9]. SV2A is the target of an anticonvulsant drug named Levetiracetam (Keppra®), and its expression has been reported to be reduced during epileptogenesis in rats, confirming the role this protein plays in epilepsy [19]. However, as previously stated, the physiopathological role of SV2A in epilepsy has not yet been clearly defined. In order to gain insight into the physiological role of SV2A, a homozygous *SV2A*-null mouse (SV2A KO) line was designed [10]. Interestingly and surprisingly, SV2A-KO mice experience severe seizures and weight loss at approximately P7, dying soon after [2, 10]. In this context, the classic knockout approach cannot be used to precisely characterise the role of SV2A, even if, once again, this protein can be linked to epilepsy.

To circumvent the limitations of SV2A-KO mice, we used a conditional gene-targeting strategy using a Cre-loxP system to produce animals that do not express SV2A in specific brain regions. In order to use the Cre-LoxP system to inactivate SV2A, we generated SV2A lox/lox mice using a gene targeting strategy. SV2A lox/lox mice exhibited a normal phenotype associated with a normal level of SV2A expression, thus rendering them indistinguishable from wild-type mice, indicating that insertion of the LoxP sites had no effect on *SV2A* gene function. When we invalidated SV2A in widespread tissues during late embryogenesis, Ubc:SV2A-cKO pups experienced postnatal growth retardation, severe seizures, and low levels of SV2A expression; the same principal defects observed in *SV2A*-null pups [10]. The only difference between our model of Ubc:SV2A-cKO mice and the SV2A-KO mice was the age of seizure onset, recorded at P12 in our model versus P7 in the SV2A-KO. This small difference could be a result of our model only invalidating SV2A at E18.5, when tamoxifen was injected, while the invalidation was already present in the early embryogenesis steps in the SV2A-KO model. All things considered, these results validated the suggestion that cre-mediated excision of the floxed SV2A allele is the cause of disruption in the *SV2A* gene function.

We then performed a zone-specific knockdown of the *SV2A* gene in the hippocampus, more precisely in the CA3 and DG regions, demonstrating that our strategy was suitable for tissue-specific targeted *SV2A* gene suppression. In order for us to be able to investigate the possible role of the SV2A protein in epilepsy, we first studied a conditional mouse line carrying *SV2A* deletion in the hippocampus (*i*.*e*., Grik4:SV2A-cKO) [14, 20]. The hippocampus is already well-known to be a generator of temporal-lobe epilepsy (TLE), a form found in humans associated with a down-regulation of SV2A expression [21]. Surprisingly, the strong reduction of SV2A protein in the CA3 hippocampal area of Grik4:SV2A-cKO mice was not associated with spontaneous seizures or reduced epileptic threshold. Several hypotheses could be formulated to explain this observation. Firstly, the strong reduction of SV2A expression observed in Grik4:SV2A-cKO mice couldn’t be sufficient to abolish the function of SV2A in the hippocampus. An investigation of the half-live of SV2A could help us to clarify this situation. Secondly, SV2A proteins are detected as early as the first 12 days of embryogenesis (E12), including in the hippocampus [18]. It is thus possible that any impact of SV2A on epileptogenesis observed at approximately P7 in an SV2A-KO mouse is more likely a consequence of SV2A, rather than a role restricted to the developmental stages of the hippocampus. Moreover, continuing with this developmental hypothesis, the expression of SV2A is modulated in the hippocampus at approximately P7, suggesting SV2A’s involvement in the early stages of development and maturation of synaptogenesis in the hippocampus [18]. The slow disappearance of the SV2A protein in the CA3 and DG regions from P14 to P56 could, in this context, not lead to epilepsy onset, as this disappearance developed too late or too slowly. Thirdly, the Grik4 promoter induced the recombination exclusively in excitatory pyramidal neurons of the CA3 area in Grik4:SV2A-cKO mice. However, SV2A-KO mouse studies have demonstrated that *SV2A* deletion results also in the reduced action potential-dependent release of the inhibitory neurotransmitter GABA in this hippocampal region. This imbalance between excitatory and inhibitory neurotransmission in the hippocampus could be responsible for seizures, at least concerning those observed in SV2A-KO mice [10, 12]. The possibility thus exists that GriK4:SV2A-cKO does not affect the neuronal population responsible for epilepsy, as the GABAergic system is preserved. Lastly, in human epileptic loci, the downregulation of SV2A expression is associated with an overexpression of SV2C in TLE patients (Crevecoeur, 2013). Therefore, the invalidation of SV2A solely in the glutamatergic system of the CA3 and DG hippocampal regions would not be sufficient to recapitulate the epileptic phenotype.

In conclusion, we have generated and validated the *SV2A flox/flox* mouse line for conditional inactivation of the *SV2A* gene after crossing with a promising Cre mouse line. This *SV2A flox/flox* mouse line will offer new opportunities to the research community to investigate the role of SV2A in specific tissues and cells.

## Supporting Information

S1 Fig Recombination efficiency and comparison of Ubc:SV2A-cKO and GriK4: SV2A-cKO. A: Relative expression of SV2A mRNA and protein of Ubc:SV2A-cKO brain and of Grik4:SV2A-cKO CA3 compared to WT (the expression of SV2A in WT mice was arbitrarily fixed at 100%). B: Ratio of SV2A Protein expression vs SV2A mRNA.
(TIFF)
