# Supplementary figures and images for: Development and Validation of a New Mouse Model to Investigate the Role of SV2A in Epilepsy
Source: PLoS One. 2016 Nov 18;11(11):e0166525. doi: 10.1371/journal.pone.0166525 (PMC5115750; doi:10.1371/journal.pone.0166525)

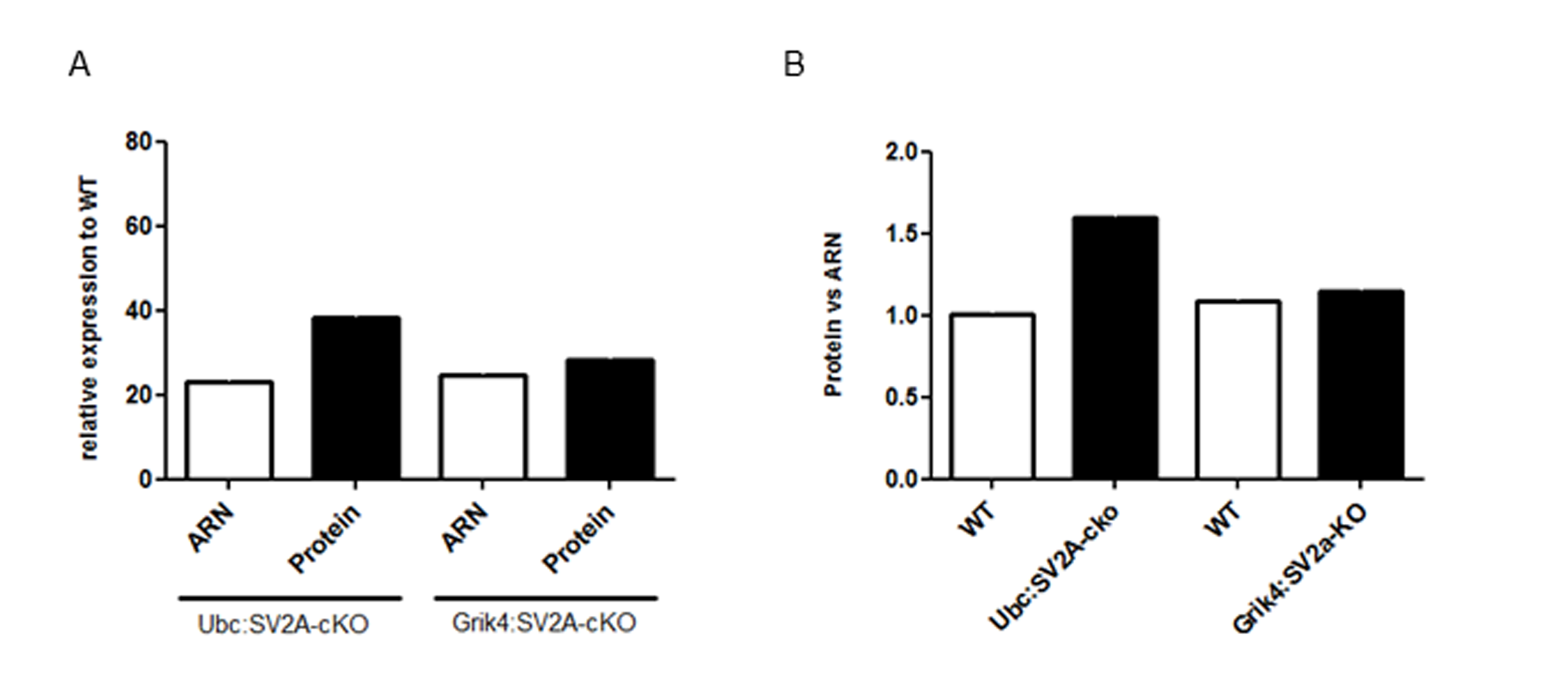

Supplement: S1 Fig — A: Relative expression of SV2A mRNA and protein of Ubc:SV2A-cKO brain and of Grik4:SV2A-cKO CA3 compared to WT (the expression of SV2A in WT mice was arbitrarily fixed at 100%). B: Ratio of SV2A Protein expression vs SV2A mRNA. (TIFF) [file pone.0166525.s001.tiff]
